# Sustainable aquaculture practices in South Asia: a comparative analysis of feed formulation and utilization

**DOI:** 10.1093/af/vfae020

**Published:** 2024-09-05

**Authors:** Shiba Shankar Giri

**Affiliations:** ICAR-Central Institute of Freshwater Aquaculture, Bhubaneswar, India

**Keywords:** aquaculture, farm-made feed, feeds, south Asian aquaculture, sustainable aquaculture

**Implications**

- South Asia practices a variety of sustainable aquaculture feed practices, including farm-made feed preparation methods for freshwater and marine fish.
- Raw material availability, cost-effectiveness, feeding regimens, and technological tools, are key factors for success.
- The varying reliance on locally sourced ingredients and the role of commercial pellet feeds reflects each country’s unique socio-economic and environmental context.
- Offering insights for policy-making and research to enhance food security and environmental conservation.

## Introduction

The fisheries and aquaculture sectors are vital in ensuring global food and nutritional security. As per FAO (2022), the global production of aquatic animals was a staggering 178 million tonnes in 2020, with aquaculture contributing a significant 87.5 million tonnes. This means that almost half of the global aquaculture animal production, at 49%, is due to the aquaculture sector. This is not just a remarkable shift but a monumental one, from the 4% share aquaculture had in the 1950s, 5% in the 1970s, 20% in the 1990s, and 44% in the 2010s. Aquaculture is not just the fastest-growing food production system globally; it has become a hope for our future food security, projected to grow at 14% by 2030 (FAO, 2022). Aquaculture plays a pivotal role in ensuring food, nutrition, and livelihood security on a global scale, which highlights its significance as a topic of utmost importance, invoking a sense of urgency and importance in our audience.

## Contribution of Fisheries and Aquaculture in South Asia

The South Asian region, comprising Afghanistan, Bangladesh, Bhutan, India, Maldives, Nepal, Pakistan, and Sri Lanka, is a powerhouse in global aquaculture production. This region produces 23.83 million tonnes of fish, representing 12.14% of the total global fish production and 14.89% of aquaculture animal production. The role of bivalves and mollusks in South Asian aquaculture is minimal, nearly nonexistent. Notably, India and Bangladesh are among the top 10 largest fish-producing nations. India is the third largest fish-producing country and the second largest in aquaculture animal production globally, with a total production of 17.545 million tonnes in 2022 to 2023, including 4.432 million tonnes from marine sources and 13.113 million tonnes from inland fisheries (DoF, Government of India, 2023). The historical development of India’s fishery sector shows a significant shift from marine-dominated fisheries to inland fisheries, which now contribute substantially to the country’s overall fish production. Currently, inland fisheries account for 75% of India’s total fish production, and within this sector, there has been a notable shift from capture fisheries to aquaculture over the past two and a half decades. Aquaculture, which constituted 34% of inland fisheries in the mid-1980s (Jayasankar, 2018), has recently surged to about 65.1% (FAO, 2024).

Bangladesh holds the third position globally in capture fisheries and the fifth in aquaculture animal production, with a total fish production of 4.75 million tonnes in 2021 to 2022. This includes 1.32 million tonnes from inland open waters (capture), 2.73 million tonnes (56.44%) from inland closed waters (culture), and 0.7 million tonnes from marine sources (DoF, Bangladesh, 2024).

Fish production in other South Asian countries is also increasing rapidly: Afghanistan produced 13,150 tonnes (FAO, 2024), Bhutan produced 195 tonnes (NDCA, personal communication, 2024), the Maldives produced 15,521 tonnes (MBS, 2023), Nepal produced 11,373 tonnes (DoL, Nepal, 2023), Pakistan produced 817,000 tonnes (PBS, 2022), and Sri Lanka produced 435,910 tonnes (Fisheries Statistics, 2022, Sri Lanka).

The growth trend in South Asian aquaculture is significantly faster than the global trend. In South Asian countries, aquaculture contributes 57.39% to the total fish production in Bangladesh, 65.01% in India, 81.54% in Nepal, and 98.97% in Bhutan. This success story of South Asian aquaculture highlights the region’s potential and opportunities for further growth and development, inspiring optimism and excitement for the future. Table 1 illustrates the fish production from various sources in South Asian countries for the year 2022.

**Table 1.** Capture fisheries and aquaculture in South Asia for the year 2022^*^

South Asian countries	Capture		Aquaculture		Total	Aquaculture, percentage of total
	Inland	Marine	Inland	BW + SW		
Afghanistan	2,000		11,150		13,150	84.8
Bangladesh	1,321,631	706,030	2,484,528		4,512,189	55.1
Bhutan			191		191	100
India	1,890,000	3,596,918	9,025,604	1,204,396	15,716,918	65.1
Maldives		155205			155205	0
Nepal	21,000		87,385		108,385	80.6
Pakistan	151,051	384,994	165,326		665,371	24.8
Sri Lanka	57,810	323,373	43,144	15,665	439,992	13.4
South Asia total	3,443,492	5,130,520	11,817,328	1,220,061	21,611,401	60.3
	Capture	8,574,012	Aquaculture	13,037,389		

Abbreviations: BW, brakish water; SW, salt water.

^*^Based on data from FAO (2024).

Over the past decade, aquaculture has grown remarkably across South Asia. In 2021, the fishery and aquaculture sector in the region provided significant employment opportunities, with 82.7% of 58.5 million people engaged full-time, part-time, occasional, or unspecified worldwide (FAO, 2022). This sector engaged 28 million people in India, 18.2 million in Bangladesh (11% of the total population), 1.12 million in Sri Lanka, 5,20,000 in Nepal, 17,589 in Maldives, and over 0.5 million in Pakistan. Additionally, more than 400 families in the southern region of Bhutan are involved in aquaculture.

The sector plays a vital role in the economies of South Asian countries, contributing 3.57% of the National Gross Domestic Product (GDP) in Bangladesh, 1.1% in India, 1.06% in Sri Lanka, and 1.0% in Nepal in 2021. In Maldives, it constitutes 98% of physical export commodities, amounting to 6% of the country’s GDP.

## Aquaculture

The majority of fish in the South Asian region are cultivated using freshwater resources. Freshwater aquaculture is primarily practiced in ponds and tanks, which can be either perennial or seasonal. The water depth in these water bodies ranges from 1.5 to 2.5 m. Additionally, freshwater aquaculture is conducted in lakes, irrigation canals, reservoirs, and paddy fields. The aquaculture production systems in South Asia are characterized as 1) extensive fish farming system: this is the least managed form of fish farming, involving pond sizes of 1 to 5 ha with a fish stocking density of less than 5,000/ha. The production rate is about 1 to 2 tonnes/ha/annum. No supplemental feeding is provided, and fish depend solely on natural food available in the water bodies. 2) Semi-intensive fish farming system: this is the most prevalent system, involving small ponds (0.5 to 1 ha) with a fish stocking density of 10,000 to 15,000 fingerlings (average size 10 grams) or 5,000 to 6,000 advanced fingerlings (average size 200 to 300 g) per hectare. The yearly production potential ranges from 3.3 to 10 tonnes/ha. 3) Intensive fish farming system: this system requires high investment and involves high stocking density and artificial feeding with mechanization. Species like pangas, tilapia, and monodons are cultivated with a production potential of 25 to 50 tonnes/ha, depending on the species. 4) Integrated aquaculture system: this system integrates aquaculture with other forms of agriculture, such as rice-fish farming, horticulture-fish farming, pig-fish farming, and poultry-fish farming.

When comparing aquaculture practices in South Asia’s two largest fish-producing countries, India and Bangladesh, distinct differences are observed. In Bangladesh, extensive, semi-intensive, and intensive systems cover 9.7%, 63%, and 28% of the total aquaculture area, respectively. In India, these systems cover approximately 35%, 60%, and 5% of the total aquaculture area.

Freshwater aquaculture in India was mainly the polyculture of Indian major carps (**IMCs**), catla (*Catla catla*), rohu (*Labeo rohita*), mrigal (*Cir. mrigala*), and exotic carps, grass carp (*Ctenopharyngodon idella*), silver carp (*Hypophthalmichthys molitrix*), and common carp (*Cyprinus carpio*). Nowadays, farmers are not very interested in growing exotic carps due to less market demand for these species. Consumers prefer the IMC species mrigal, but farmers are less interested in growing it due to its difficulty harvesting. The present practice is bispecies aquaculture, in which 80% to 90% of the population is shared by rohu and 10% to 20% by catla. Over the period, other important and minor carps such as *L. fimbriatus*, *L. gonius*, *L. bata*, *Oxygaster spp*, *Rasbora spp*, *Cir. cirrhosa*, *Puntius kolus*, *P. carnaticus*, *P. pulchellus*, *P. sarana*, *P. sophore*, *P. ticto*, *Amblypharyngodon mola,* freshwater butter catfish (*Ompok bimaculatus*), and Amur carp (*Cyp. rubrofuscus*) are also considered as candidate species for aquaculture since they have consumers’ demand. IMCs contribute about 75%, and all carp species constitute about 90% of the total freshwater fish production in the country. Species like *Pangasianodon hypophthalmus* (Sutchi Catfish) have made inroads into Indian aquaculture, with an annual production of 0.5 million tonnes. Similarly, *Wallago attu*, *Mystus seenghala*, *M. aor*, *Pangasius pangasius*, *Rita pavimentata*, *Clarias batrachus*, *Heteropneustes fossilis, Anabas testudineus,* and murrels (*Channa striata* and *Ch. marulius*) are in great demand in eastern and north-eastern states of India. The culture of high-valued freshwater prawn species, *Macrobrachium rosenbergii* and *Mac. malcolmsonii*, is gaining popularity. India has significant cold water fishery resources in upland streams/rivers in the Himalayan and Western Ghats. Commercial farming of rainbow trout (*Oncorhynchus mykiss*) in raceways has gathered momentum in some Himalayan states.

Brackishwater aquaculture in India primarily focuses on *Penaeus monodon* (Tiger shrimp). Lately, the cultivation of the exotic species *Leptopenaeus vannamei* (White leg shrimp) has surged and now dominates over 80% of *Pen. monodon* farming. However, India’s vannamei production contracted sharply in 2023 (possibly by 12%), but its monodon production continues to expand (Jory, 2023). In 2019, vannamei and monodon shrimp production was less than 6,00,000 tonnes. Their production rose to 6,50,000 tonnes in 2020; in 2021, the volume escalated to 9,30,000 tonnes. In 2022, the production declined to 9,02,525 tonnes because of low farm gate prices of shrimp, high input costs in farming, insurgency of diseases, and nonavailability of credit for shrimp farming (AquaAsiaPac., 2024). Recently, open sea cage farming of cobia (*Rachycentron canadum*), silver pompano (*Trachinotus blochii*), and Milkfish, *Chanos chanos* is gaining traction, along with seaweed cultivation.

Inland aquaculture resources in Bangladesh mainly include ponds, ditches, baors, shrimp and prawn farms, and seasonal water bodies. Inland aquaculture of indigenous and exotic carp, tilapia, pangas (*Pan. hypophthalmus*), and Vietnam koi (*A. testudineus*) expanded massively throughout the country. Besides, new interest grew in farming indigenous species viz., koi (*A. testudineus)*, singh (*Het. fossilis*), magur (*Cla. magur*), pabda (*O. pabda*), gulsha (*M. cavasius*), mola (*A. microlepis*), etc. because they are getting scarce on open waters but have high market demand. Floodplain aquaculture is very popular in Bangladesh and is practiced with a community-based approach. The emergence of exotic species aquaculture is a relatively recent development in Bangladesh and has assumed increasing importance since the late 1990s after the import of exotic pangasius *Pangasianodon hypophthalmus* broodfish from Thailand in 1993. Subsequently, the introduction of single-sex tilapia hatcheries and advancements in spawning methods for various lesser-known commercial species like the climbing perch *A. testudineus*, stinging catfish *Het. fossilis*, and walking catfish *Clarias spp* opened up new avenues in Bangladesh’s aquaculture. In coastal aquaculture, shrimp/prawn and finfish farming is expanding and complying with good aquaculture practices. Nowadays, ecofriendly integrated farming is also becoming more popular. As many as 35 aquaculture technologies are in practice now in Bangladesh in inland and coastal waters. The notable one is *Pen. monodon* monoculture in coastal waters. Shrimp and prawn production increased from 160 thousand tonnes in 2002 to 2003 to 2,51,964 tonnes in 2020 to 2021, with a total export volume of 30,571 tonnes and a value exceeding USD 407.25 million in 2021 to 2022 (DoF, Bangladesh, 2023).

In Pakistan, the inland aquaculture assets encompass various water bodies, such as ponds, lakes, reservoirs, ditches, flood compartments, abandoned canals, and seepage ponds. Among these, pond-based fish farming stands out as the most prevalent method. Traditionally, IMCs like rohu (*L. rohita*), mori (*Cir. mrigala*), and thaila (*C. catla*), along with Chinese carps silver carp, grass carp, common carp, and more recently, bighead carp (*H. nobilis*), have been favored species. Trout farming is concentrated in the Northern Himalayan valleys, including Swat, Hazara Division, Murree, Kaghan, and Azad Kashmir. Additionally, there is a growing interest in Mahseer, Sol (*Ch. marulius*), and Singhari (*M. singhala*) aquaculture. GIFT Tilapia has been introduced on a small scale in aquaculture operations. Carp polyculture systems, both semi-extensive and extensive, are prevalent in warmer waters, while in colder regions, trout farming is conducted intensively using flow-through concrete raceways.

In Sri Lanka, freshwater aquaculture mainly consists of freshwater food fish culture in seasonal tanks and earthen ponds. In addition, freshwater giant prawn (*Mac. rosenbergii*) culture is also practiced in seasonal tanks and earthen ponds. Brackish water aquaculture mainly dominates the culture of the black tiger prawn (*Pen. monodon*). Although other species like milkfish, Asian Seabass, grouper, mud crab, mussel, oyster, and sea cucumber show promise, large-scale commercial cultivation is still developing. Asian seabass (*Lates calcarifer*) and mud crab (*Scylla serrata*) culture in earthen ponds, seabass culture in floating net cages and pens in lagoons and sea cucumber culture in pens in lagoons are the recent aquaculture practices. Under an agreement between Vietnam and the National Aquaculture Development Authority (**NAQDA**) of Sri Lanka, expertise from Vietnam is utilized for sea cucumber breeding in a private hatchery. At the same time, NAQDA oversees sea cucumber cultivation in pens and ponds. Seabass farming in cage culture is on the rise in Trincomalee Bay. Around 80 farmers were involved, approximately 67,210 seabass seeds were stocked, and 27.24 tonnes were harvested in 2015. Milkfish fry and fingerlings are readily available in coastal and brackish water areas, with the Mannar tidal flats estimated to produce about 4 million fingerlings annually. Some farmers are involved in mud crab (*S. serrata*) fattening in cages or ponds. Approximately 90% of ornamental fish production originates from aquaculture, with only about 10% being wild caught. Common ornamental fish species include guppies, swordtails, platys, barbs, tetras, angels, gouramis, and catfish. Freshwater ornamental fish culture is practiced for export purposes and to meet local needs (Athukorala, 2017).

Aquaculture production is relatively recent in the Maldives compared to the rest of the Asia-Pacific region. Recently, the Government of Maldives recognized the potential of the aquaculture industry, mainly based on high-valued marine food fish, targeting export markets. The major thrust species are sea cucumber, sandfish (*Holothuria scabra*), brown-marbled grouper (*Epinephelus fuscoguttatus*), and milkfish (*Cha. chanos*). Only one commercial aquaculture venture is in operation in Maldives, and it has an annual production of approximately 144 tonnes of sandfish. A Maldivian Government initiative to develop mariculture enterprises, with the assistance of the International Fund for Agricultural Development, is now working on extending aquaculture grow-out operations at the community level. In addition, small-scale grow-out operations for hatchery-produced groupers and the hatchery production of milkfish as an alternative live feed for the pole-and-line fishery are expected to kick off soon (Ahmed, 2017).

Nepal, Bhutan, and Afghanistan are the land-locked South Asian countries. In Nepal, the species used in aquaculture are common carp (*Cyp. carpio*), silver carp (*H. molitrix*), big head carp (*Aristichthys nobilis*), grass carp (*Ctenopharyngodon idella*), rohu (*L. rohita*), naini (*Cir. mrigala*), bhakur (*C. catla*). Three types of aquaculture systems adopted by the carp farmers, viz. low-density carp polycultures with yearlings, high-density carp polycultures using fingerlings, and chhari production (50 to 75 g size) by stocking naini and rohu fry at high density. In the low-density system, carp yearlings (40 to 150 g) are stocked at a density of 7,150 to 8,000 fish/ha, while in the high-density system, fingerlings (3 to 25 g) are stocked at a density of 15,400 to 18,500 fish/ha. The Charri production system exclusively involves *Cir. mrigala* (Naini) and *L. rohita* (Rohu), with fry-sized fish (1 to 5 g) stocked at a density of 63,000 to 76,000/ha. Regardless of the aquaculture practices, 2.2 to 7.3 tonnes of cow dung and 40 to 170 kg of inorganic fertilizers are used in 1-ha pond yearly. Very recently, Tilapia (*Tilapia niloticus*), Pangas (*Pangasius* spp), and Rainbow trout (*Onc. mykiss*) have been introduced into aquaculture. Development in cold water aquaculture started to take momentum after the accomplishment of the import of rainbow trout from Japan in 1988. At present, rainbow trout farming is done in raceways in 18 districts. Production volumes in low-density aquaculture systems range from 5.63 to 7.38 tonnes/ha using pellet feed. Production is low in a high-density aquaculture system, ranging from 4.86 to 5.32 tonnes/ha, as the fish are grown with mash feed. The Chhari fish culture system recorded the highest production of 7.91 tonnes/ha with mash feeds (Prasad, 2017).

Non-native carp are the mainstay of Bhutan’s aquaculture. Although most of the Bhutani population are fish eaters, they do not prefer aquaculture because of their religious background. About 400 households in the southern part of the country regularly engage in aquaculture and fisheries activity. The species cultured are grass carp, silver carp, common carp, rohu, mrigal, and catla. Bhutan’s annual output of farmed fish production is less than 200 tonnes (Drukpola, 2017).

Afghanistan’s government drafted a fisheries and aquaculture policy in 2015, but aquaculture has yet to be picked up fully.

## Aquafeeds

### Global status

Aquaculture relies heavily on feed, which typically accounts for 50% to 80% of total production costs (FAO, 2017). Not only does feed play a significant role in cost, but it also profoundly influences the quality, safety, and nutritional value of farmed fish. The quantity and quality of feed needed vary based on species’ feeding habits, physiological stages, and environmental conditions like temperature and availability of natural food.

According to Alltech’s 2023 Agri-Food Outlook, aqua feed constitutes approximately 4.2% (52.9 million tonnes) of the global compound feed production, totaling 1.266 billion tonnes in 2022. The report indicates a 2.7% increase in global aqua feed production within the aquaculture sector. China, Vietnam, India, Norway, and Indonesia have emerged as leading aquaculture feed producers.

Data presented in Alltech Agri-Food Outlook 2023 from over 140 countries and 2,800 feed mills highlights China (260.739 million tonnes), the United States (240.403 million tonnes), Brazil (81.948 million tonnes), India (43.360 million tonnes), and Mexico (40.138 million tonnes) as the top 5 feed-producing countries. Together, these nations contribute 64% of the world’s feed production. Notably, China, the United Staes, Brazil, and India collectively consume half of the world’s feed (Mehr, 2023). The aquafeed production in different regions of the world is presented in Table 2. The Asia-Pacific region stands out as the primary global aqua feed producer, experiencing a production growth of over 2.6% in 2022. Altech’s data indicate a rise in aqua feed production from 37.3 million tonnes in 2021 to 38.3 million tonnes in 2022, attributed to China’s economic resurgence and increased standards of living, which have boosted demand for aquatic products. Countries like the Philippines, Bangladesh, South Korea, and Malaysia have also witnessed growth in feed production. Fed aquaculture currently constitutes about 75% of global aquaculture production, with over 90% of China’s aquaculture production relying on feed-based methods.

**Table 2.** Aquafeed production by region (million tonnes)

Region	2021	2022	Growth (%)
Africa	1.484	1.449	−2.38
Asia-Pacific	37.350	38.340	2.65
Europe	4.605	4.687	1.78
Latin America	5.652	5.922	4.79
Middle East	0.500	0.566	13.14
North America	1.730	1.750	1.16
Oceania	0.190	0.200	5.26
Total	51.510	52.914	2.72

Source: Yildez (2023).

### South Asian status

In South Asia, the primary method of aquaculture production for finfish and crustaceans is through semi-intensive earthen pond farming systems. Most of these systems, particularly those focusing on freshwater noncarnivorous finfish, representing over 80% of total finfish production in Asia, rely on supplementary feeding with farm-made feeds. The productivity of natural ponds significantly contributes to the nutrient requirements of these species, thereby reducing feed costs. Three feeding practices are commonly observed in South Asian aquaculture: (i) the use of industrially produced pelleted feed, (ii) the use of a combination of industrial and farm-made feed mixes, and (iii) the use of on-farm farm-made feeds composed of locally available feed ingredients. Carp and omnivores are typically fed a mixture of bran and cake with a feed conversion ratio (**FCR**) ranging from 2.5 to 3.5 in semi-intensive aquaculture systems (Giri, 2017). Industrially compounded feeds for fish and shellfish generally have a lower FCR, ranging from 1.2 to 1.6. When selecting feed ingredients for aquafeeds, three main factors are considered: (1) quality, which includes nutrient composition and the presence of antinutrients or contaminants that may hinder nutrient absorption; (2) quantity, ensuring a sufficient and consistent supply; and (3) price of ingredients. Other challenges in fish feed management encompass feed formulation, processing, storage, handling, and transport.

**Figure 1.**
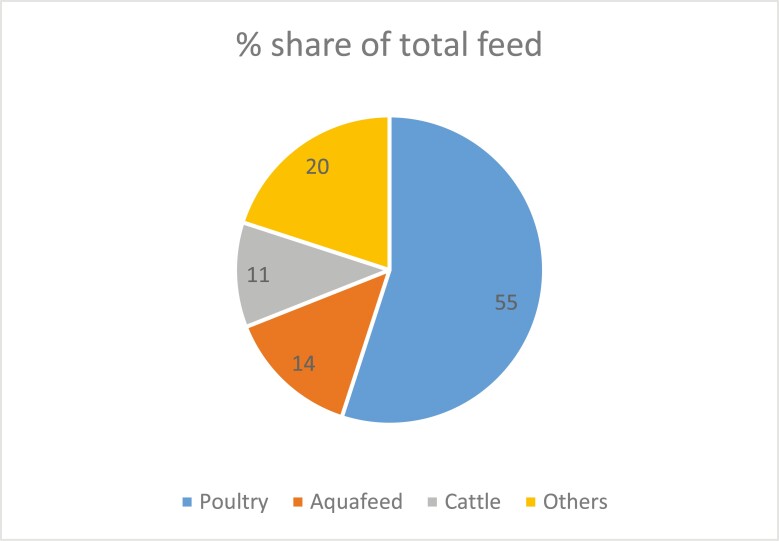
Share of the Indian animal feed market. Source: Mehr (2023).

At least 80% of aquafarmers in India currently use some supplementary feed, indicating that traditional extensive aquaculture is disappearing fast. The country produced 44 million tonnes of compound feed for all the feed user sectors in 2021 (Figure 1) and experienced a decrease of 1.6% in 2022, producing 43.4 million tonnes. Despite the decline, the country managed to rank fourth in the world compound feed production in 2022 (Yildiz, 2023). The total palleted aquafeed production in India in 2021, excluding output from small feed millers and toll milling, ranged from 2.0 to 2.37 million tonnes. There are 38 high-tech feed plants, with a total production capacity of 3.5 million tonnes, manufacture palleted aquafeeds. The country imports at least 1.5 million tonnes of pelleted aquafeeds annually (FASAR, 2015) and also exports about 1 million tonnes to neighboring countries. In 2019, the volume of shrimp feed deals was assessed at 1.3 million tonnes (Dinesh and Durai, 2021). In 2021, India’s farmed shrimp production rose to 920,000 tonnes. Demand for shrimp feed increased by 7% to 10% in 2021 to reach 1.35 million tonnes, fueled partly by the shift of fish farms to vannamei shrimp farming in low salinity conditions (AquaAsiaPac, 2024). The retail price for shrimp feed is approximately $1.10 to $1.40/kg. Farmers paying cash are given a 10% to 15% discount, dealer discounts are usually 10% to 15%, and the feed mill profit is 10% to 15%. The remaining one million tonnes of feed was used in finfish aquaculture to produce 0.65 million tonnes of fish, assuming 1.5 FCR. Our estimation shows that in 2022, the Indian aquaculture sector used 20 million tonnes of feed, of which 6.16 million tonnes compounded feed, including 2.37 million tonnes of pelleted feed and 13.95 million tonnes of farm-made feed. In pond aquaculture, the FCR is the apparent measure derived from the amount of feed introduced into the pond system and the net biomass of fish produced. The FCR of compounded feeds varies from 1.2 to 2.0, whereas the average FCR for farm-made feed is 3.0.

Recently, there has been a significant increase in the cost of formulating shrimp feeds, rising from 7% to 35%. This contrasts with the relatively stable cost of fish feed, which slightly increased from INR 2 to 3/kg. The spike in shrimp feed costs is attributed mainly to the elevated prices of key ingredients such as soybean meal (**SBM**), fishmeal, and wheat flour. Conversely, the cost drivers of fish feeds are deoiled rice bran (**DORB**), rice bran, corn, dried distillers grains and solubles, deoiled groundnut cake, and deoiled mustard cake.

Interestingly, while fish prices have been on a downward trend for an extended period, especially since the onset of the Covid-19 pandemic’s second wave, many farmers are now opting for more economical and lower-specification feeds. Instead of premium feeds with higher protein and lipid content, they turn to economy brands containing 20% to 24% crude protein and 3% lipid. Farmers have even discontinued using pelleted feeds in some areas, shifting towards methods utilizing low-cost DORB and mash feeds.

In 2020, nearly all farmers in Bangladesh (95%) used supplementary feeds in aquaculture. Among those, 59% used formulated and nonformulated types, with only 4% exclusively using formulated feed. Also, 37% of total feed usage consisted of formulated feed, while the remaining 63% comprised nonformulated options. Notably, floating feed accounted for 22% of the overall feed usage (Ali et al., 2023). The average rate of feed use in 2020 was 3.25 tonnes/ha, an increase of 30% to 38% since 2013 (Jahan et al., 2015; Ali et al., 2016). In Bangladesh, the number of feed producers, importers, and retailers is proliferating with the expansion of the aquaculture industry. Feed production increased from 0.8 to 1.5 million tonnes between 2013 and 2016, while demand increased from 1.8 to 2.5 million tonnes over the same period. Around 186 feed mills produce commercial aquafeeds, among which 10 to 15 large operators account for 60% to 70% of the market share (Rashid et al., 2013). Almost all feed mills also produce poultry feeds. Meanwhile, 15 companies have installed extruders in their factories. The most common types of feeds in use are nursery feeds, usually in the form of mash, and starter feeds, which are in the form of mash or crumble. Depending on fish size, grower and finisher feeds, as pellets, are fed to fish. The main culture period of commercial fish is between March and November; therefore, the highest volumes of aquafeed are produced during April and September. The share of total feeds varies among cultivable species: pangasius 60% to 65%, tilapia 35% to 45%, climbing perch 10% to 15%, carp and others 2% to 5%, and shrimp and prawn 2% to 3%. The categories of feeds also vary according to the production phases: nursery phase 2% to 3%, starter feed 20% to 30%, and grower/finisher feed 60% to 70%. Extrusion technology is mainly used to produce floating feed for tilapia, accounting for around 40% to 50% of total tilapia feed production. The main raw materials used for aquafeeds and their inclusion levels are rice bran 20% to 50%, maize 5% to 20%, SBM 10% to 20%, mustard oil cake 10% to 25%, fishmeal 5% to 15%, and meat and bone meal 10% to 20% (Rashid et al., 2013).

In Pakistan, aquaculture does not involve the practice of feeding with compounded feeds. Carps are the dominant aquaculture species, and they are grown at low stocking density. Fish are fed rice bran in mash form. The concept of artificially manufactured feed is very new, and it was introduced in the country with all-male tilapia culture technology. The production of tilapia has reached 15,000 tonnes in the current year. It is becoming more popular daily and encouraging the adoption of intensive farming and artificial fish feed. Only two fish feed manufacturing factories operate in Pakistan on a very small scale, manufacturing feed for carp, tilapia, catfish, and shrimp. The major feed ingredients are very similar to those used for poultry feed, e.g., fish meal, SBM, canola meal, sunflower meal, and gluten (Amman et al., 2020).

In Nepal, approximately 12,000 tonnes of pellet feed are utilized in aquaculture, with one-third imported from India and the remainder locally produced. Despite being costlier, floating pellets are favored for trout and pangasius farming. About 60% of carp species are fed a combination of pellets and farm-made feed. In comparison, roughly 40% of small-scale carp farmers rely solely on natural pond productivity, occasionally aided by fertilizers (NACA, 2017). Rice bran, oil cakes, and wheat flour are common ingredients and are used as mash feeds. Commercial pellet feeds are slowly replacing mash feed, with 13.3% of farmers using a combination of pellets and mash feed and 20% using pellets exclusively. Trout and Pangas farmings are completely pelleted feed-based.

The feed industry in Sri Lanka primarily focuses on producing poultry and cattle feed, with imports covering the demand for shrimp feeds and specific ornamental fish feeds. Ornamental fish farmers use locally manufactured as well as imported feeds for their operations. However, feeds for other aquaculture practices, such as freshwater food fish culture, seabass culture, and crab culture, are not imported because of their high costs. Instead, farmers often use poultry feeds for food fish culture and may occasionally prepare aquafeeds by blending poultry feeds with fish meal and rice bran (Athukorala, 2017). For ornamental fish farming, live feeds are commonly used during the early stages of fish growth, while pellet feeds, whether local or imported, are preferred for later life stages. Conversely, feeding is not practiced in seasonal tanks used for freshwater food fish culture. These tanks, which are relatively shallow yet highly productive, host omnivorous fish species *Oreochromis mossambicus*, *O. niloticus*, *C. catla*, *Cir. mrigala*, and *L. rohita*. In the seasonal tanks, these fish subsist on natural feeds such as phytoplankton, zooplankton, algae, aquatic insects, aquatic macrophytes, and detritus (Pushpalatha et al., 2021).

While aquaculture feed demand in the Maldives is confined to small-scale research endeavors, it is projected to expand as the country’s aquaculture sector develops. Despite the absence of domestic aquaculture feed production due to limited local demand, there exists potential for its development in the Maldives. Fishmeal derived from tuna processing byproducts, constituting approximately 50% to 60% of processed animals’ weight, is produced and primarily exported to Sri Lanka. Recent export data indicates an annual volume of around 1,000 tonnes of fish meal, priced at roughly USD 1/kg. Initial assessments suggest that with minor enhancements in ash content, locally produced fish meal holds promise as a protein source in aquaculture feeds. Anticipated growth in local demand for aquaculture feed, particularly for cultured groupers and milkfish, is foreseen with the establishment of a multispecies hatchery aiming to produce 100 tonnes of juvenile milkfish for local pole-and-line fishing and approximately 500,000 grouper fingerlings for small-scale grow-out operations within the Maldives. Although initially sourced internationally, aquaculture feeds for hatchery production, it is expected that local production of more cost-effective feeds will emerge as the number of grow-out farmers increases (Naeem, 2017).

## Opportunities in Aquafeed Market and Industry

- Rising disposable incomes, evolving culinary tastes, and exposure to global cuisines among South Asians fuel increased fish consumption, indicating optimistic prospects for the regional feed market.
- Fish and fish products are perceived as healthier, more nutritious, and cost-effective than other animal proteins, propelling growth in the aquaculture feed sector.
- Despite a rise in per capita fish consumption in South Asia, it remains among the lowest globally, presenting opportunities for industry consolidation and market expansion.
- The aquaculture sector in the region has transitioned from small-scale backyard operations to large-scale, technology-driven farming, emerging as a profitable and well-organized industry. Despite this growth, substantial aquafeeds are currently imported to meet the industry’s expanding needs. However, given that the South Asian region is tropical, significant alternative sources of aquafeed ingredients are available locally.
- The shrimp segment has expanded to fulfill demands in export markets and is projected to continue its growth trajectory.
- The aquafeed industry, predominantly producing traditional and decentralized feeds, presents an opening for new entrants to introduce more organized and enhanced commercial feed options, shifting the industry landscape.

## Challenges in the Aquafeed Sector

- Rapid aquaculture expansion strains marine and terrestrial feed resources, worsened by competition from animal husbandry, poultry, and human food sectors, resulting in elevated feed prices.
- Ongoing exploration of alternative lipid and protein sources for aquafeeds displays potential but intensifies pressure on terrestrial feed resources.
- Despite progress, fishmeal and fish oil retain their critical status as feed ingredients due to their superior nutritional value, acceptance by fish, and human health benefits.
- Developing nutrient requirement tables and feeding standards for all cultivable species at different life stages and within various culture systems is a very challenging task but is crucial for the judicious use of feed in aquaculture.
- Adherence to the FAO Code of Conduct for Responsible Fisheries is vital for exporting aquaculture products, particularly concerning fishmeal and fish oil from wild fisheries.
- Concerns about the misuse of antibiotics, hormones, and chemicals in aquafeeds undermine consumer trust in fish and fishery products.
- Implementing technological interventions in feed processing to improve feed utilization presents another avenue for reducing feed costs in aquaculture.

## Farm-Made Aquafeeds and Feeding Practices in South Asia

Carps are the mainstay of the South Asian Aquaculture, characterized by their omnivorous feeding habits and reliance on plant-based feeds. They are typically cultivated in semi-intensive earthen pond polyculture systems, where supplementary feeding is common across the region. The natural productivity of these ponds is sustained by applying animal and poultry excreta and chemical fertilizers.

Many small-scale and marginal farmers cultivate carp in backyard ponds, a form of extensive aquaculture system, often without providing additional feed. Moreover, many of these farmers are unaware of the necessity of feed for fish growth. In South Asia, the vast majority (over 95%) of carp are raised using farm-made feeds, typically comprising simple mixtures of one or two ingredients without adhering to balanced nutrition. Local feed ingredients are utilized (Table 3). These ingredients normally contain less than 10% moisture and their chemical compositions are presented in Table 4. They are used in different combinations in farm-made feeds, administered as mash, dough, or occasionally pellets.

**Table 3.** Ingredients used in farm-made feeds in South Asian aquaculture

Name of the country	Ingredients used in farm-made aquafeeds
India	Rice bran, deoiled rice bran, wheat bran, maize bran, tapioca flour, mustard oil cake, sunflower oil cake, cotton seed cake, til oil cake, copra cake, linseed oil cake, lin seed sludge, kitchen waste
Bangladesh	Rice bran, wheat bran, mustard oil cake, sunflower oil cake, til oil cake, kitchen waste. Slaughterhouse byproducts
Nepal	Rice bran, wheat bran, soybean meal, mustard oil cake.
Sri Lanka	Rice bran, deoiled rice bran, maize bran, mustard oil cake, coconut oil meal.
Pakistan	Rice bran, rice middling, wheat bran, cassava leaves, kitchen refuse, oil seed cakes
Bhutan	Rice bran, maize grain, maize grit, molasses, mustard oil cake.
Afghanistan	—
Maldives	Tuna processing wastes, fish gutted wastes, wheat bran

**Table 4.** Proximate composition (on DM basis) of the ingredients used for farm-made aquafeeds

Source	CP%	EE%	CF%	Ash%
Rice bran	12 to 16	12 to 14	8 to 12	5 to 8
Deoiled rice bran	15 to 18	1 to 2	10 to 15	8 to 12
Wheat bran	11 to 14	2 to 3	10 to 12	2 to 3
Molasses	3			10
Corn meal	9.5	4	3.8	1.7
Tapioca flour	3 to 6	2 to 3	2 to 6	2 to 3
Mustard oil cake	25 to 38	6 to 9	10 to 16	10 to 12
Soybean oil cake	44 to 48	1 to 2	4 to 5	5 to 6
Til oil cake	35 to 42	3 to 6	10 to 15	10 to 13
Cotton seed acke	32 to 35	6 to 8	12 to 16	8 to 12
Sunflower oil cake	30 to 32	4 to 8	15 to 18	8 to 10
Copra cake	20 to 25	6 to 10	12 to 17	5 to 8
Coconut oil meal	20 to 25.9	11.2 to 11.4	16.2 to 17.9	6.2 to 8.9
Lin see oil meal	30 to 32	6 to 10	12 to 15	10
Linseed sluge	4.4	19.1		
Brewer’s yeast	44.2	1.4	3.0	6.8
Slaughterhouse wastes	54 to 65	9 to 26	—	5 to 9

Abbreviations: CP, crude protein; EE, ether extract/crude fat; CF, crude fiber.

Source: Paul et al. (2017) ; Rath et al. (2014); Mohanty et al. (2013); Ayyappan and Ahamad Ali (2007).

In 2022, farm-made aquafeed production in India amounted to approximately 13.95 million tonnes. Typically, a ratio of 2.5 to 3.5 kg of farm-made feed, averaging at 3 kg, is utilized for producing one kg of carp. In polyculture settings, carp are fed DORB, oil cake, or a combination of the two, often in a 10:90 cake-to-bran mixture. Initially, carp juveniles are exclusively fed DORB until they reach a body weight of 350 g, after which a mixture of DORB and oil cake is introduced.

During the 8-month grow-out period, farmers typically hang 8 to 10 perforated bags containing 5 to 8 kg of mash feed each in an acre of pond water area for daily feeding. Farmers also sometimes use moist feed balls made from farm-made feeds, placing them in plastic or bamboo feeding trays suspended from bamboo poles within the pond. These trays are positioned to prevent contact with the pond bottom, thus deterring feeding by crabs and snails. Some farmers employ small machinery to produce farm-made feed pellets, which are sun-dried and stored in gunny or plastic bags for later use. Familiar sources of oil cake include mustard, groundnut, til, linseed, and cottonseed cakes, while animal proteins are not used in farm-made feeds. Farm-made feeds do not typically contain vitamin and mineral supplements unlike commercial feeds. The micronutrient requirements of fish are being met through the pond environment. Farmers in the Krishna-Godavari delta, known as the fish basket of India, annually use 760 kg of single super phosphate, 440 kg of urea, 30 kg of diammonium phosphate, and 220 kg of potash per hectare of pond area. These inorganic fertilizers help grow and sustain plankton populations in the ponds. The inorganic fertilizers and unutilized feeds support the micronutrient pool in the water and soil. The guiding principle behind farm-made aquafeeds in India is using locally sourced, primarily unconventional, and low-cost ingredients. Furthermore, the seed production of carp, pangasius, and tilapia also largely relies on farm-made feeds, predominantly mixtures of rice bran and oil cake, without compounded feeds.

From 2008 to 2018, the Indian Council of Agricultural Research (**ICAR**) initiated an outreach initiative titled “Fish Feeds” in a collaborative network involving Research Institutes and fish farmers. The overarching goal of this project was to tackle critical challenges in developing and managing quality feeds, aiming to bolster the production of prioritized fish and shrimp species to meet India’s escalating demand. The objectives were tailored to foster the creation of live and formulated farm-made feeds to improve fish survival and growth. Additionally, the project sought to devise feed management strategies synchronized with biological rhythms for grow-out fish culture while exploring avenues for enhanced feed utilization (Giri, 2017).

Over a dozen farm-made feeds were meticulously formulated and tailored for various freshwater, brackish water, marine water, and cold water species, including cage culture setups. These feeds were accessible to farmers across different sectors to bolster aquaculture practices and encourage adoption. An inventory detailing the availability and chemical compositions of feed ingredients nationwide was compiled, facilitating the preparation and publication of feed formulations utilizing locally abundant and cost-effective ingredients (Mohanty et al., 2013). Moreover, protocols were established to produce live foods and farm-made feeds efficiently.

The outreach effort encompassed numerous nationwide awareness programs, leveraging mass media, classroom instruction, and exposure visits to disseminate knowledge. Skill development initiatives, such as hands-on training and participatory demonstrations, were organized to empower fish farmers. As a result of utilizing farm-made feeds, farmers experienced a notable increase in carp production fourfold, 4 to 6 tonnes/ha/yr, achieving a FCR of 1.5 to 1.6, compared to their earlier rates of production from 1.0 to 1.5 tonnes/ha (Rath et al., 2014; Paul et al., 2019). Farmers also learned to harness locally available ingredients to reduce production costs substantially, diminishing reliance on traditional supplementary feeds like groundnut cake and rice bran mixtures.

In Bangladesh, aquaculture mostly depends on farm-made feeds for carp and tilapia. These feeds are formulated based on the availability and cost of raw materials, with various methods used for preparation, including grinding, mixing, and extrusion. Despite commercial pellet feeds, 0.3 to 0.4 million tonnes of farm-made pelleted feeds are produced in approximately 1,000 small pellet mills operating in the country. These small pellet-preparing machines are crafted locally, which cost about 100,000 to 250,000 BDT (1,250 to 3,125 USD). These mills can produce feed pellets ranging from 50 to 300 kg/hr and are usually operated by small-scale farmers or feed traders (Hossain, 2017). However, operators face challenges such as inadequate pellet binding and drying and a lack of knowledge in formulating feeds that meet the nutritional needs of fish at different growth stages. Some commercial feed mills also offer rental services to large-scale farmers, adjusting their formulations and pricing based on local purchasing capacity. However, concerns exist regarding the quality and performance of feeds produced in these facilities due to limited formulation knowledge and inconsistent supply and quality of ingredients.

In Nepal, the preparation and processing of farm-made fish feed have not been extensively developed. According to a survey (Wagle et al., 2017) conducted in nine districts of the southern Terai region of Nepal, most farmers rely solely on mash feed for fish production, with a smaller percentage using a combination of mash and pelleted feed or pellets alone. Some farmers use single ingredients such as oil cake, rice bran, brewery byproducts, and bakery products as feed sources. Rice bran emerges as the most commonly used ingredient (96%), followed by oil cake (84%) and wheat flour (41%) in mash feeds. Many farmers remain unaware of commercial pellet feeds and show little interest due to their perceived high costs. Regardless of the fish’s life stages, farmers feed carp at least once daily. A preference for feeding larvae three times a day is observed among 43% of farmers, while 29% still opt for once-a-day feeding. Most farmers (65%) adopt a two-time feeding regimen for fry rearing, while over 70% feed fingerlings and grow-out fish once daily. Morning feeding is common among farmers, primarily for its convenience. Generally, hand-feeding is the preferred method across all fish sizes, although some farmers utilize feed trays and bags for feeding larvae and table-sized fish. The mean FCR for mash feed, comprising 40% oil cake and 60% rice bran, are recorded at 2.42:1, with the highest FCR observed in rice bran alone at 3.40:1.

Farmers in rural Sri Lanka engage in freshwater food fish cultivation, utilizing backyard earthen ponds and relying on locally accessible, cost-effective raw materials such as rice bran, broken rice, coconut oil meal, dried fish offal, cooked poultry offal, kitchen waste, expired bread, shrimp feed, and poultry feed to nourish the fish. Some farmers opt for a blend of poultry feed, rice bran, coconut oil cake, and locally sourced fishmeal powder as fish feed. Farmers commonly employ cow dung and lime to augment natural food production in the ponds. Among small-scale seabass farmers, the utilization of trash fish or fish offal as feed is prevalent due to its profitability, albeit challenged by difficulties in sourcing these ingredients during lean periods. Simple and economical tools are employed for feed preparation, such as electric kitchen grinders for small-scale grinding tasks. Grinding larger quantities necessitates the use of grinding mill services. Various sizes of mesh sieves are available for sieving raw materials into fine powder, with the choice dependent on the quantity to be sieved. Conventional kitchen scales are utilized to measure feed ingredients. Occasionally, farmers use steamers to precook feed dough before pelletization. Depending on the scale of pellet feed production, equipment such as string hopper makers, manual meat mincers, or electric meat mincers are employed.

In the Maldives, current aquaculture efforts focus solely on tiger grouper and sandfish. Tiger grouper cultivation is being conducted on a trial basis, with feed demands remaining modest due to limited aquaculture output. The feed regimen for tiger groupers involves providing tuna meat sourced from discarded tuna leftovers from processing facilities at a rate of 3% of the fish’s body weight every other day. The cost of discarded tuna meat is approximately USD0.33/kg. Farm-made feed preparation involves utilizing locally sourced fish meals and deceased seagrass gathered from coastal areas. Additionally, imported ingredients such as SBM, rice bran, vitamin premix, and mineral premix are incorporated into the feed formulation (Naeem, 2017).

## Outlook

Exploring and identifying alternative indigenous feed resources, conducting their nutrient analyses, ameliorating antinutritional factors, and formulating region-specific, cost-effective farm-made feeds can reduce feed costs and bridge future aquafeed demands. Developing nutrient requirement tables and dietary and feeding standards for all cultivable species at various life stages and culture systems represents a practical approach to promoting judicious feed utilization in aquaculture. Establishing guidelines for farm-made feed processing, manufacturing, handling, packaging, labeling, and on-farm storage is essential to maximize feed utilization and minimize wastage.

Moreover, it is crucial to develop guidelines for standardized feed management and feeding practices and appropriate equipment for feed dispensing in aquaculture. However, the real game-changer lies in educating large fish farmers on the benefits of using pellet feeds. This can aid in better resource management, addressing future challenges of feed scarcity and reducing sediment deposition in ponds for clean water fish farming. Similarly, empowering small and marginal farmers with knowledge on the importance of feeding in aquaculture and training them in preparing farm-made feeds using locally available, cost-effective ingredients can significantly enhance production and farm returns.

Enhancing natural pond productivity can also reduce feed usage and production costs. However, it is not just about reducing costs but also about ensuring the safety and effectiveness of the feeds. This is where the establishment of South Asian Regional Referral Laboratories for aquafeed quality assurance and certification comes into play. By promoting good management practices in aquaculture and enforcing harmonized feed regulations in the region, we can take vital steps to ensure the quality and safety of manufactured feeds.

Fostering inter-institutional linkages and capacity building through education, research, training, exchange programs, and exposure visits among stakeholders in SAARC Member States can further encourage regional fish production. Implementing a uniform feed policy and regulation for all sectors of resource users, including animal husbandry, poultry, and aquaculture, will incentivize fish farmers to engage in feed-based aquaculture.

## About the Author

 **Dr. Shiba Shankar Giri** leads the Fish Nutrition and Physiology Division at ICAR-Central Institute of Freshwater Aquaculture in Bhubaneswar, India. His research spans aquaculture nutrition, fish physiology, nutrigenomics, farm-made aquafeeds, and feeding standards for aquatic animals. With over 200 research papers, 20 book chapters, and a monograph to his credit, Dr. Giri has also edited 10 aquaculture books for the South Asian Association for Regional Cooperation (SAARC). He spent 3 years as the Senior Program Specialist (Fisheries) at the SAARC Agriculture Centre in Dhaka, Bangladesh. Additionally, Dr. Giri has served as an editor for several journals, including Reviews in Aquaculture, SAARC Journal of Agriculture, Fishes, and the Indian Journal of Animal Nutrition. He is a Fellow of the National Academy of Veterinary Science (India) and the Animal Nutrition Society of India. **Corresponding author**: ssgiri1965@gmail.com
